# Comparison of the short-term efficacy and safety of bipolar transurethral electro vaporization and holmium laser enucleation of the prostate for moderate and large benign prostatic enlargement

**DOI:** 10.1186/s12894-023-01215-8

**Published:** 2023-03-29

**Authors:** Yutaro Hayashi, Shuko Yoneyama, Akitoshi Takizawa, Kazuki Kobayashi, Hiroki Ito

**Affiliations:** 1 Department of Urology, Yokosuka Kyosai Hospital, 1-16, Yonegahama douri, Yokosuka, Kanagawa Japan; 2 Department of Urology, Kokusai Shinzen Sougou Hospital, 1-28-1, Nishigaoka, Izumi-ku, Yokohama, Kanagawa Japan; 3 Department of Urology, Yokohama City University Graduate School of Medicine, Kanagawa, Japan

**Keywords:** Vaporization, Laser enucleation, Benign prostatic enlargement (BPE), Lower urinary tract symptoms (LUTS), Bipolar

## Abstract

**Background:**

To compare the efficacy and safety of bipolar transurethral vaporization of the prostate (B-TUVP) with holmium laser enucleation of the prostate (HoLEP) for moderate [prostate volume (PV) 30–80 ml] and large (≥ 80 ml) benign prostatic enlargement (BPE).

**Materials and methods:**

Male patients with lower urinary tract symptom (LUTS) or urinary retention who underwent B-TUVP or HoLEP treatment in two regional centers were respectively enrolled. Patient characteristics and treatment outcomes were retrospectively compared between B-TUVP and HoLEP.

**Results:**

In patients with moderate and large prostate volume,B-TUVP showed shorter operative time (P < 0.001) and less hemoglobin decrease (P < 0.001) than in HoLEP. In uncatheterised patients, voiding symptoms and patients’ quality of life improved after B-TUVP and HoLEP, but these improvement rates were consistently bigger in HoLEP than in B-TUVP. In catheterised patients, the rate of achieving catheter-free status after surgery was higher in HoLEP than in B-TUVP for patients with PV > 80 ml.(P < 0.001) The incidence of postoperative fever was higher in B-TUVP than in HoLEP for patients with PV 30–80 ml (P < 0.001) but not for those with PV > 80 ml.(P=0.08) The Incidence of postoperative stress incontinence(SUI) was higher in HoLEP than in B-TUVP for patients with moderate and large prostate volume.

**Conclusions:**

There are few studies that investigated the short-term efficacy and safety of second-generation B-TUVP in comparison with HoLEP for moderate and large BPE. Improvement in LUTS and achievement of catheter-free status were predominant in HoLEP, and these outcomes were more prominent in patients with large BPE of PV > 80 ml. However, B-TUVP resulted in less blood loss, shorter operative duration, and less SUI suggesting that B-TUVP is also well-tolerated surgical modality.

**Supplementary Information:**

The online version contains supplementary material available at 10.1186/s12894-023-01215-8.

## Introduction

Surgical treatment is one of the treatment opition for benign prostate enlargement (BPE). Monopolar Transurethral resection of the prostate (M-TURP) has long been considered as the gold standard for the surgical management of BPE. However, in recent years various techniques such as HoLEP (holmium laser enucleation of the prostate) and B-TUVP (bipolar transurethral vaporization fo the prostate) have been developed with the aim of providing a safe and effective alternative to M-TURP.

HoLEP has broader indications for its use than B-TUVP. It is currently recommended for the treatment of moderate (PV 30–80 ml) and large (PV > 80 ml) BPE [1, 2]. A previous paper did not support the long-term advantage of TUVP over TURP for BPE treatment [3, 4]. However, we have several reasons for performing B-TUVP instead of TURP including favorable learning curve, lower costs, better safety profile in anticoagulated patients on the basis of our previous papers [5, 6].

Recently, we introduced the second-generation B-TUVP using an oval electrode (certification number: 29ABBZX00048000,26th Jun. 2017, Olympus, Japan) and demonstrated its efficacy and safety for large BPE (PV ≥ 100 ml) [5]. Our findings revealed that the second-generation B-TUVP facilitated more efficient vaporization owing to the wider surface of its oval electrode.

This confirmed the hypothesis that B-TUVP is an alternative treatment option for HoLEP in patients with moderate and large BPE. A previous study [7] suggested that conventional B-TUVP was comparable with HoLEP for small BPE less than 40 ml. In spite of this, there is a dearth on literature comparing the efficacy and safety of B-TUVP versus HoLEP in men with moderate BPE (PV 30–80 ml) and large (PV ≥ 80 ml) BPE.

This current study was a retrospective review of two regional centers that compared the efficacy and safety of second-generation B-TUVP against HoLEP in patients with moderate and large BPE, with the aim of evaluating and eventually establishing the future outlook of B-TUVP for BPE treatment.

## Materials and methods

### Study population

After obtaining Institutional Review Board study protocol approval, we retrospectively compared clinical data from two regional centers employing second-generation B-TUVP (Yokosuka Kyosai Hospital) and HoLEP (Kokusai Shinzen Sougou Hospital) for BPE treatment. Both hospitals are located in the same district, Kanagawa, in Japan.

This study enrolloed 161 consecutive patients with BPE who underwent B-TUVP in Yokosuka Kyosai Hospital and 286 consecutive patients who underwent HoLEP in Kokusai Shinzen Sougou Hospital. Because of no records of preoperative PV, 4 patients from the B-TUVP group and 5 patients from the HoLEP group, were excluded. The B-TUVP procedure was performed by eight senior urologists and HoLEP by seven senior urologists. The mean age of urologists (34.4 ± 9.4 and 35.8 ± 7.8 years-old in Yokosuka Kyosai Hospital and Kokusai Shinzen Hospital, respectively) were almost equal and thus we believed that seniority of urologists in 2 hospitals were not so different.

All the B-TUVP and HoLEP procedures in this study were performed between July 2018 and October 2021 at Yokosuka Kyosai Hospital and between September 2016 and March 2021 at Kokusai Shinzen Sougou Hospital. Abdominal ultrasound or prostate MRI scans were employed to calculate PV with measurement of 3-dimensional diameter of the prostate.

The two centers shared the same surgical indications and management of elevated prostate-specific antigen (PSA) value and anticoagulant/antiplatelet therapy, which were described in previous studies [5]. Briefly, surgical indications were International Prostate Symptoms Score (IPSS) > 7, maximum flow rate (Q_max_) < 10 ml/s, persistent or recurrent urinary retention or bladder stones. The patients with PSA ≥ 4 ng/ml were recommended to undergo prostate MRI and if that indicated suspected prostate cancer, they were counseled to undergo needle biopsy before prostate surgery. If patients were receiving anticoagulant/antiplatelet therapy during the perioperative period, anticoagulant/antiplatelet therapy was temporarily discontinued before prostate surgery, under the physicians’ and anesthetists’ guidance; medication was resumed after confirming the absence of hematuria. If the physicians recommended retaining anticoagulant/antiplatelet therapy, we informed the patient thoroughly of the possible higher risks of perioperative bleeding before proceeding with prostate surgery. If the patient decided to undertake prostate surgery despite the higher risk of bleeding, they underwent prostate surgery as a standard surgical procedure under anticoagulant/antiplatelet therapy. In the TUVP group with PV > 80 ml, 2 patient were operated on under continuous anticoagulant/antiplatelet therapy, and in the HoLEP group, all patients had stopped anticoagulant/antiplatelet therapy.

The B-TUVP and the HoLEP patient characteristics and treatment outcomes were then retrospectively compared. Hemoglobin (Hb) levels were measured preoperatively and on the first postoperative day (POD). Intraoperative blood loss was estimated by hemoglobin changes. Hb change (%) was calculated by (Hb at 1POD / preoperative Hb) x100. Operative time was defined as duration of endoscopic procedures. The main treatment outcomes were measured by IPSS and IPSS Quality of Life Index (IPSS-QoL) at preoperative baseline, then at 1 and 3 postoperative months (POM). IPSS change (%) and IPSS QoL change (%) were calculated by (values at 1 or 3POM / preoperative values) x 100.

The study was conducted in accordance with local regulations and principles set out in the Declaration of Helsinki. The study protocol (IRB number YKH21-69 and KSSH3219_07) was approved by the institutional ethics committee of Yokosuka Kyosai Hospital and Kokusai Shinzen Sougou Hosptial. Informed consent was obtained in the form of an opt-out on the Yokosuka Kyosai Hospital and Kokusai Shinzen Sougou Hospital.

### Operative procedures of B-TUVP and HoLEP

Operative procedures of B-TUVP were described previously [5]. Briefly, under spinal or general anesthesia, the patient was placed in the lithotomy position and sterile draped. A 26 Fr continuous-flow resectoscope (30° cystoscopic lens) was inserted into the bladder and an oval electrode (Olympus, Japan) was used for vaporization of the prostate. Irrigation with normal saline was performed using a 26 Fr resectoscope, utilizing the transurethral resection in saline (TURis) system by Olympus and operating on cutting/coagulation settings at 200 W/120 W. After completing vaporization and coagulation, an 18 Fr three-way transurethral catheter was installed, only to be removed when hematuria had resolved (approximately on 2–4 POD). [3].

Alternatively, HoLEP was performed using a 120 W holmium YAG laser (VersaPulse PowerSuite, Lumenis Surgical, San Jose, CA, USA) with a 550 nm end-firing fiber (SlimLine, Lumenis). A 26 Fr continuous-flow resectoscope with saline irrigation was used. The laser settings were at 2.5 J and 40 Hz. After enucleation of the adenoma and control of bleeding, the enucleated adenomas were removed from the bladder using a mechanical tissue morcellator (Versa-Cut, Lumenis) with an indirect nephroscope. For the unexpected bleeding after enucleation, the monopolar TUR was deployed to coagulate in HoLEP.

The preoperative antibiotic, a third- generation cephalosporin, was administered before surgery and on PODs 1 and 2 in both B-TUVP and HoLEP.

### Statistical analysis

Statistical analysis was undertaken using SPSS software (SPSS version 22, Inc., Chicago, IL). Paired and un-paired student’s t test and Mann–Whitney U test were used, as appropriate, to compare the preoperative and postoperative continuous variables between the groups. The chi-square and Fisher’s exact tests were used to compare discrete variables. Continuous variables were presented as mean and standard deviation, and discrete variables were presented as percentages. P values < 0.05 were considered significant.

## Results

Table 1 summarized the patient characteristics and surgical outcomes of patients with PV 30–80 ml.

The mean age for B-TUVP is higher than HoLEP .The mean Prostate volume is not significantly different between B-TUVP and HoLEP. In uncatheterised patients from both the B-TUVP and HoLEP groups, total IPSS and IPSS-QoL scores significantly decreased after surgery (at 1 and 3 POM) compared to baseline within each group (P < 0.001). Between B-TUVP and HoLEP groups, total IPSS was significantly lower in HoLEP than in B-TUVP at 1 and 3 POM. In catheterised patients, the rate of achieving catheter-free status after surgery was relatively higher in HoLEP than in B-TUVP but this was not statistically significant. A total of 2 cases in the B-TUVP group required a 2nd procedure for persistent LUTS after the initial session.

**Table 1:** Patient characteristics and surgical outcomes of bipolar transurethral electro vaporization of the prostate (B-TUVP) and holmium laser enucleation of the prostate (HoLEP) in patients with prostate volume of 30–80 ml

		B-TUVP	HoLEP	P value *
All patients		N=75	N=166	
Age(years-old)		76.4 ± 6.8	71.5 ± 7.4	<0.001
Prostate volume (ml)		57.3 ± 14.8	56.0 ± 14.2	0.5
Operative time (mins)		93.7 ± 22.8	125.3 ± 36.7	<0.001
Duration of post-operative catheterisation (days)		2.6 ± 1.1	2.3 ± 1.9	0.3
Hospital stay period after operation (days)		5.9 ± 2.4	6.7 ± 2.5	0.018
Hemoglobin (g/dl)	Change (%)	96.0 ± 1.4	90.4 ± 5.7	<0.001
Necessity of 2nd procedure		2 (2.7%)	0	0.034
Uncatheterised patients		N = 45	N = 135	
Total IPSS	Pre	22.3 ± 7.2	22.1 ± 7.2	0.9
	1POM	13.3 ± 7.9	9.3 ± 6.3	0.004
	Change (%) at 1POM	69.3 ± 43.3	45.1 ± 32.7	0.008
	3POM	9.8 ± 7.4	6.7 ± 5.2	0.018
	Change (%) at 3POM	46.0 ± 34.8	36.3 ± 39.2	0.1
IPSS-QoL	Pre	5.2 ± 1.0	4.8 ± 1.1	0.09
	1POM	3.5 ± 1.8	3.0 ± 1.6	0.1
	Change (%) at 1POM	69.1 ± 39.9	68.6 ± 53.3	0.7
	3POM	2.6 ± 1.7	2.1 ± 1.5	0.1
	Change (%) at 3POM	53.0 ± 32.9	37.1 ± 30.0	0.06
Catheterised patients		N = 30	N = 31	
Achieving catheter free status after surgery		20 (67%)	27 (87%)	0.06

*Compared between B-TUVP and HoLEP.

Values were presented as mean ± SD or number of cases (%).

Hb change (%) was calculated by (Hb at 1POD / preoperative Hb)x100.

IPSS change (%) and IPSS-QoL change(%) were calculated by (values at 1 or 3POM / preoperative values) x 100.

The patient characteristics and surgical outcomes of B-TUVP and HoLEP in patients with PV > 80 ml are summarized in Table 2. The mean age and prostate volume are same in two groups.

In uncatheterised patients in both B-TUVP and HoLEP groups, total IPSS and IPSS-QoL scores significantly decreased after surgery (at 1 and 3 POM) compared to baseline within each surgery group (P < 0.001). Between B-TUVP and HoLEP, total IPSS and IPSS-QoL were significantly lower in the HoLEP group than in the B-TUVP group at 1 and 3 POM. In catheterised patients, the rate of achieving catheter-free status after surgery was significantly higher in HoLEP than in B-TUVP. A total of 8 cases of B-TUVP required 2nd procedures to address persistent urinary retention, whereas 3 cases of HoLEP had to undergo repeat sessions owing to incomplete morcellation.

**Table 2:** Patient characteristics and surgical outcomes of bipolar transurethral electro vaporization of the prostate (B-TUVP) and holmium laser enucleation of the prostate (HoLEP) in patients with prostate volume > 80 ml

		B-TUVP	HoLEP	P value *
All patients		N=82	N=115	
Age(years-old)		74.4 ± 7.5	73.0 ± 6.6	0.1
Prostate volume (ml)		119.4 ± 44.9	111.2 ± 29.8	0.1
Operative time (mins)		120.28 ± 31.0	164.6 ± 43.0	<0.001
Duration of post-operative catheterisation (days)		3.0 ± 1.4	2.5 ± 1.2	0.009
Hospital stay period after operation (days)		6.8 ± 2.7	7.0 ± 2.8	0.2
Hemoglobin (g/dl)	Change (%)	96.0 ± 6.5	84 ± 10.6	<0.001
Necessity of 2nd procedure		8 (9.8%)	3 (2.6%)	0.03
Uncatheterised patients		N = 48	N = 76	
Total IPSS	Pre	20.3 ± 8.6	22.3 ± 8.4	0.295
	1POM	13.8 ± 8.2	8.8 ± 6.2	0.001
	Change (%) at 1POM	86.0 ± 63.9	50.4 ± 64.2	< 0.001
	3POM	10.6 ± 8.0	6.0 ± 5.0	0.002
	Change (%) at 3POM	62.2 ± 42.0	27.0 ± 20.0	< 0.001
IPSS-QoL	Pre	4.9 ± 1.1	4.7 ± 1.3	0.581
	1POM	3.7 ± 1.7	2.5 ± 1.8	0.005
	Change (%) at 1POM	78.5 ± 33.0	58.5 ± 72.2	0.005
	3POM	2.9 ± 1.8	1.7 ± 1.5	0.002
	Change (%) at 3POM	63.3 ± 39.0	30.7 ± 30.8	0.001
Catheterised patients		N = 34	N = 39	
Achieving catheter free status after surgery		24 (71%)	39 (100%)	<0.001

* Compared between B-TUVP and HoLEP.

Values were presented as mean ± SD or Number of cases (%).

Hb change (%) was calculated by (Hb at 1POD / preoperative Hb)x100.

IPSS change (%) and IPSS-QoL change(%) were calculated by (values at 1 or 3POM / preoperative values) x 100.

Table 3 displays the summary of postoperative surgical complications in both TUVP and HoLEP groups. The incidence of postoperative fever was significantly higher in B-TUVP than in HoLEP in patients with a PV 30–80 ml but not in PV > 80 ml. There were two cases of septic shock requiring catecholamine in addition to hydration and broad-spectrum antimicrobial therapy and one postoperative cerebral cortex infarction (Clavien-Dindo Grade IVb) in a B-TUVP (PV > 80 ml) patient. Postoperative stress urinary incontinence and complications associated with morcellation occurred only in the HoLEP group and all cases were treated conservatively (Clavien-Dindo Grade I), however, 2 of 24 postopertive stress urinary incontinence were presistent 6month follow-up.　The other 22 postoperative stress urinary incontinence was transient and ceased without any treatment within 6 months.

Both hospitals experienced a case in which transurethral coagulation was necessary due to concerns about catheter obstruction and prolonged hematuria with progression of anemia. No blood transfusion was required and no transurethral resection (TUR) syndrome occurred in both surgical groups.

**Table 3:** Surgical complications of B-TUVP and HoLEP in patients with prostate volume of 30–80 ml and > 80 ml

		B-TUVP	HoLEP	P value *
PV 30-80ml	Number of cases	75	166	
	Post operative fever	14 (19%)	4 (2.4%)	<0.001
	Bladder tamponade †	1 (1.3%)	0	0.1
	Prostate capsule injury	0	1 (0.6%)	0.5
	Bladder neck perforation	0	2 (1.2%)	0.3
	Urethral stricture	1 (1.3%)	5 (3.0%)	0.4
	Post operative SUI §	0	9 (5.4%)	0.04
PV > 80ml	Number of cases	82	115	
	Post operative fever	15 (18%)	11 (10%)	0.08
	Bladder tamponade †	0	2 (1.7%)	0.2
	Bladder injury	0	2 (1.7%)	0.2
	Prostate capsule injury	0	1 (0.9%)	0.4
	Prostate neck perforation	0	1 (0.9%)	0.4
	Urethral stricture	0	1 (0.9%)	0.4
	Post operative SUI §	0	15 (13%)	<0.001

*Compared between B-TUVP and HoLEP

† Bladder tamponade was defined as massive blood clot in the bladder causing voiding difficulty.

§ Post operative stress urinary incontinence was defined as pad usage of at least 1 per day.

## Discussion

Shorter operative times, and reduced hemoglobin changes were seen in the B-TUVP group than in the HoLEP group in both PV 30–80 ml and > 80 ml. In contrast, a shorter duration of postoperative catheterisation and a reduction in the necessity for a 2nd procedure were observed more in the HoLEP group than in the B-TUVP group, especially in patients with PV > 80 ml. In uncatheterised patients, voiding symptoms and patients’ QoL derived from IPSS and IPSS-QoL clearly and significantly improved after B-TUVP and HoLEP in both PV 30–80 ml and PV > 80 ml, but these improvement rates were consistently greater in HoLEP than in B-TUVP. In catheterised patients, the rate of achieving catheter-free status after surgery was significantly higher in HoLEP than in B-TUVP for patients with PV > 80 ml and PV 30–80 ml. The incidence of postoperative fever was significantly higher in B-TUVP than in HoLEP in patients with PV 30–80 ml but not in PV > 80 ml.

Retrospective studies and systematic review compared conventional B-TUVP with TURP suggesting better hemostatic efficiency, similar short-term functional outcomes, shorter operative and catheterization duration, shorter hospital stays, and fewer postoperative complications for moderate BPE (PV < 80 m)l [8–11]. Meanwhile, prospective randomized trial showed that long-term efficacy and the safety of plasmakinetic vaporization of prostate was not not comparable to the results after TURP [3], and another RCT showed that major disadvantage of B-TUVP were the lack of a tissue specimen, relatively high retreatment rate and less effectiveness compared with TURP [4]. Besides, a few previous studies compared B-TUVP not only TURP but also laser vaporization. A randomized controlled trial showed no difference in symptom control between B-TUVP and GreenLight laser photoselective vaporization of the prostate (GL-PVP) for moderate BPE (30–80 ml) at 2 years [12]. The greater cost of GL-PVP compared with B-TUVP was an important concern. Unfortunately, none of those studies were suitable for meta-analysis [13].”

Meanwhile, several other randomized controlled trials comparing HoLEP with various BPH treatment modalities including TURP, photoselective vaporization of the prostate (PVP), and open prostatectomy, demonstrated the high efficacy and safety of HoLEP over other treatment modalities [14–19]. In general, systematic reviews and meta-analyses demonstrated that there is long-term improvement in voiding function even for large BPH (PV > 80 ml) after HoLEP [13, 20, 21]. However, HoLEP is associated with a steep learning curve, with longer operating time and difficulty in the enucleation procedure seen as the most crucial problems for a beginner [22].

Both B-TUVP and HoLEP exhibited their efficacy through high rates of postoperative catheter-free status in this study. Particularly, for catheterised patients with PV > 80 ml, the postoperative catheter-free rate was significantly higher in HoLEP than in B-TUVP with a similar trend seen in patients with PV 30–80 ml. These findings indicated that HoLEP has clinical advantages over B-TUVP in achieving catheter-free status for patients with urinary retention, especially in patients with PV > 80 ml. B-TUVP may have less of an advantage in producing this outcome as it sometimes results in inadequate vaporization and residual adenoma compared to HoLEP in PV > 80 ml.

The most common surgical complication of both B-TUVP and HoLEP was postoperative fever which was more frequent in B-TUVP than in HoLEP, especially in patients with PV 30–80 ml. Vaporization via B-TUVP dilates blood vessels possibly leading to higher risks of infection than enucleation with HoLEP. One of the general drawbacks of HoLEP was its prerequisite for morcellation during surgery which subsequently leads to serious operative complications including bladder injury. Furthermore, the rate of postoperative stress urinary incontinence was significantly higher in HoLEP than in B-TUVP. Transient and mixed urinary incontinence was well-documented as a major complication of HoLEP, with incidence rates at 4.3–16.2% [23–27]. Most case of stress urinary incontinence were reported as transient and, in this study, almost stress urinary incontinence was transient and ceased without any treatment within 6 months. Only two postopertive stress urinary incontinence were observed 6month follow-up.

The rate of hemoglobin reduction was lower in B-TUVP than in HoLEP suggesting that the bipolar system has a higher hemostatic capacity than holmium laser.

Limitations of the current study include its retrospective nature and the short duration of follow-up. The small number of enrolled patients may mean it was underpowered for some parameters in statistical analysis. However, the results of this study were consistent with clinical data from two regional centers designated for each surgical modality; thus we believe that the data quality we collected was enough to achieve the aim of this study. A randomized trial with a longer follow-up period will be necessary to confirm the long-term efficacy of B-TUVP.

## Conclusions

There are few studies that investigated the short-term efficacy and safety of second-generation B-TUVP in comparison with HoLEP for moderate and large BPE. For both uncatheterised and catheterised patients, improvement in LUTS, achievement of catheter-free status, and the non-necessity of a 2nd procedure were predominant in HoLEP, and these outcomes were more prominent in patients with large BPE of PV > 80 ml. However, B-TUVP resulted in less blood loss, shorter operative duration, and less urinary incontinence in both moderate and large BPE suggesting that B-TUVP is a well-tolerated surgical modality.

## Electronic supplementary material

Below is the link to the electronic supplementary material.

Additional File: The original data set used in this study

## Data Availability

The raw data underlying this paper is available without any restriction in the anonymous manner as a supporting information.

## List of Abbreviations

AUA: American Urological Association
BPE: Benign prostatic enlargement
BPH: Benign prostatic hyperplasia
B-TUVP: Bipolar transurethral vaporization of the prostate
Hb: Hemoglobin
EAU: European Association of Urology
HoLEP: Holmium laser enucleation of the prostate
IPSS: International Prostate Symptom Score
IPSS-QoL: IPSS Quality of Life Index
LUTS: Lower urinary tract symptoms
N.S.: Not significant
OABSS: Overactive Bladder Symptom Score
PVP: Photoselective vaporisation of the prostate
PVR: Post-void residual urine volume
POD: Postoperative day
POM: Postoperative months
PV: Prostate volumes
PSA: Prostatic-specific antigen
Qmax: Maximum uroflow rate
SUI: Stress urinary incontinence
TURP: Transurethral resection of the prostate
